# Involuntary Retirement and Depression Among Adults: A Systematic Review and Meta-Analysis of Longitudinal Studies

**DOI:** 10.3389/fpsyt.2022.747334

**Published:** 2022-02-04

**Authors:** Long Zhai, Junhui Wang, Yantao Liu, Hua Zhang

**Affiliations:** ^1^Department of Occupational Health, Qingdao Municipal Center for Disease Control and Prevention, Qingdao Institute of Prevention Medicine, Qingdao, China; ^2^Department of Third Supervisory Section, Shibei District Health Supervision Institute, Qingdao, China; ^3^Department of Chronic Noncommunicable Diseases, Qingdao Municipal Center for Disease Control and Prevention, Qingdao Institute of Prevention Medicine, Qingdao, China

**Keywords:** depressive symptom, epidemiology, meta-analysis, retirement, work

## Abstract

Results from longitudinal studies on involuntary retirement and depression remain controversial. PubMed, Web of Science, Embase, ScienceDirect, Wanfang, and VIP updated on 4 January 2022 were searched for eligible publications. Pooled relative risks (RRs) with 95% confidence interval (CI) were calculated using a random-effects model. Eight published articles with 14,604 participants for the effect of involuntary retirement on depression incidence and 26,822 participants for the relationship between depression and involuntary retirement were included. Compared with working, the pooled RR for depression was 1.31 (95% CI, 1.13–1.51; *I*^2^ = 37.7%) for the involuntary retirement overall. For involuntary retirement, the pooled RR was 1.70 (95% CI, 1.28–2.25; *I*^2^ = 84.2%). The associations between involuntary retirement and depression did not substantially change in sensitivity and subgroup analyses. No evidence of publication bias was found. This meta-analysis indicates that there might be mutual causal relationship between involuntary retirement and depression. More large longitudinal studies with different gender and income levels are needed.

## Introduction

Depression is a prevalent chronic condition which often leads to increased morbidity and functional impairment; more than 264 million people are living with depression all around the world (1). Depression predisposes to medical illnesses and advances biological aging. Medical illnesses also increase the risk of late-life depression. The reciprocal relationships of depression with aging-related and disease-related processes have generated pathogenetic hypotheses (2).

Retirement is a major life transition in the second half of life with changes in daily schedules and social activities. Moving from a relatively busy and regular lifestyle to a relatively inactive one is a challenge, and the change of roles may be a trigger for some retirees to develop mental health problems (3).

Involuntary retirement was defined as being forced to retire before the regular retirement age due to business closure, layoff, family problems, or health problems (4). During the past couple of decades, studies did not reach a consensus on the relationship between involuntary retirement and depression (5–12). For the effect of involuntary retirement on depression incidence, a longitudinal cohort study reported that unemployed was not associated with the risk of depressive symptoms (10). However, another longitudinal study showed involuntary retirement increased the risk of depressive symptoms (9). Other longitudinal studies about the association between depression and involuntary retirement have also provided conflicting results. One nationally representative panel survey indicated that depression and depressive symptoms were significantly associated with retirement in late middle-aged U.S. workers (7). Whereas, a Chinese longitudinal study showed that depression had no effect on involuntary retirement (12).

Therefore, we conducted a meta-analysis of longitudinal studies to: (1) research the causal relationship between involuntary retirement and depression; (2) explore the potential between-study heterogeneity and (3) investigate the potential publication bias.

## Methods

This systematic review was written according to the Preferred Reporting Items for Systematic Reviews and Meta-Analyses (PRISMA) guidelines (http://www.prisma-statement.org).

### Literature Search and Selection

The English literatures of PubMed, Embase, ScienceDirect, and Web of Science and the Chinese literature of Wanfang, and VIP from their establishment to 4 January 2022 will be comprehensively and systematically searched. PubMed, Embase, ScienceDirect, and Web of Science were searched through the subject words and keywords retrieval method using the following keywords: “retirement” and “depression”. The Wanfang, and VIP were searched using the general Chinese translation of the above search terms (Table S1). Moreover, we reviewed the reference lists from retrieved articles to search for further relevant studies.

The eligibility criteria were mainly conducted in accordance with the PICOS (population, intervention/exposure, control, outcomes, and study design) principle limited to Chinese and English study.

The inclusion standards were shown below: (a) Population. Community participants; (b) Exposure. Employment status or depression state; (c) Comparators. Healthy adults (age ≥ 19) in normal working condition; (d) Outcomes. Depression or early retirement; (e) Study design. Longitudinal study.

The following exclusion criteria were utilized: (a) papers which were meta-analysis, reviews, animal experiments, case reports, conference abstracts, non-English/Chinese literature, mechanism researches or other diseases, or lacking the full text; (b) duplicate publication or incomplete data; (c) study provided insufficient information on multivariate-adjusted RRs/ORs of retirement and depression; (d) participants already taking antidepressants or having hospital treatment for depression.

### Data Extraction and Quality Assessment

The following data were extracted from each study by two investigators: (1) name of the first author; (2) publication year; (3) study population; (4) origin of country; (5) follow-up years; (6) number of participants; (7) age range or mean age at baseline years; (8) gender; (9) measurement of employment status and depression; (10) RR/OR with 95% CI (adjusted by the most confounders in the original studies); (11) adjustment for confounders; (12) study quality. The study quality was assessed using the Newcastle-Ottawa quality assessment scale (http://www.ohri.ca/programs/clinical_epidemiology/oxford.asp).

### Statistical Analysis

We weighted the study-specific log relative risks by the inverse of their variance to calculate a summary estimate and its 95% CI. The DerSimonian and Laird random effects model was used to combine study-specific effect sizes (95% CIs), which considers both within-study and between-study variation (13). *I*^2^ of Higgins and Thompson was used to assess heterogeneity among studies (14) and *I*^2^ values of 0, 25, 50, and 75% represent no, low, moderate and high heterogeneity (13), respectively.

Univariate meta-regression analyses by study region, number of participants, and follow-up years were conducted to investigate the potential sources of heterogeneity. The leave-one-out sensitivity analysis (15) was carried out to evaluate the key studies that have a substantial impact on the between-study heterogeneity.

Publication bias was assessed with visual inspection of the funnel plots, Begg's rank correlation test (16), and Egger's linear regression test (17). We also conducted subgroup analyses by study region (Europe, America, and Asia), number of participants (≥4,000 and <4,000), and depression measurement (Center for Epidemiologic Studies Depression scale). All statistical analyses were conducted by Stata V.12.0 (Stata Corp, College Station, Texas, USA). A two tailed *p* < 0.05 was considered statistically significant.

## Results

### Literature Search and Study Characteristics

**Figure 1:**
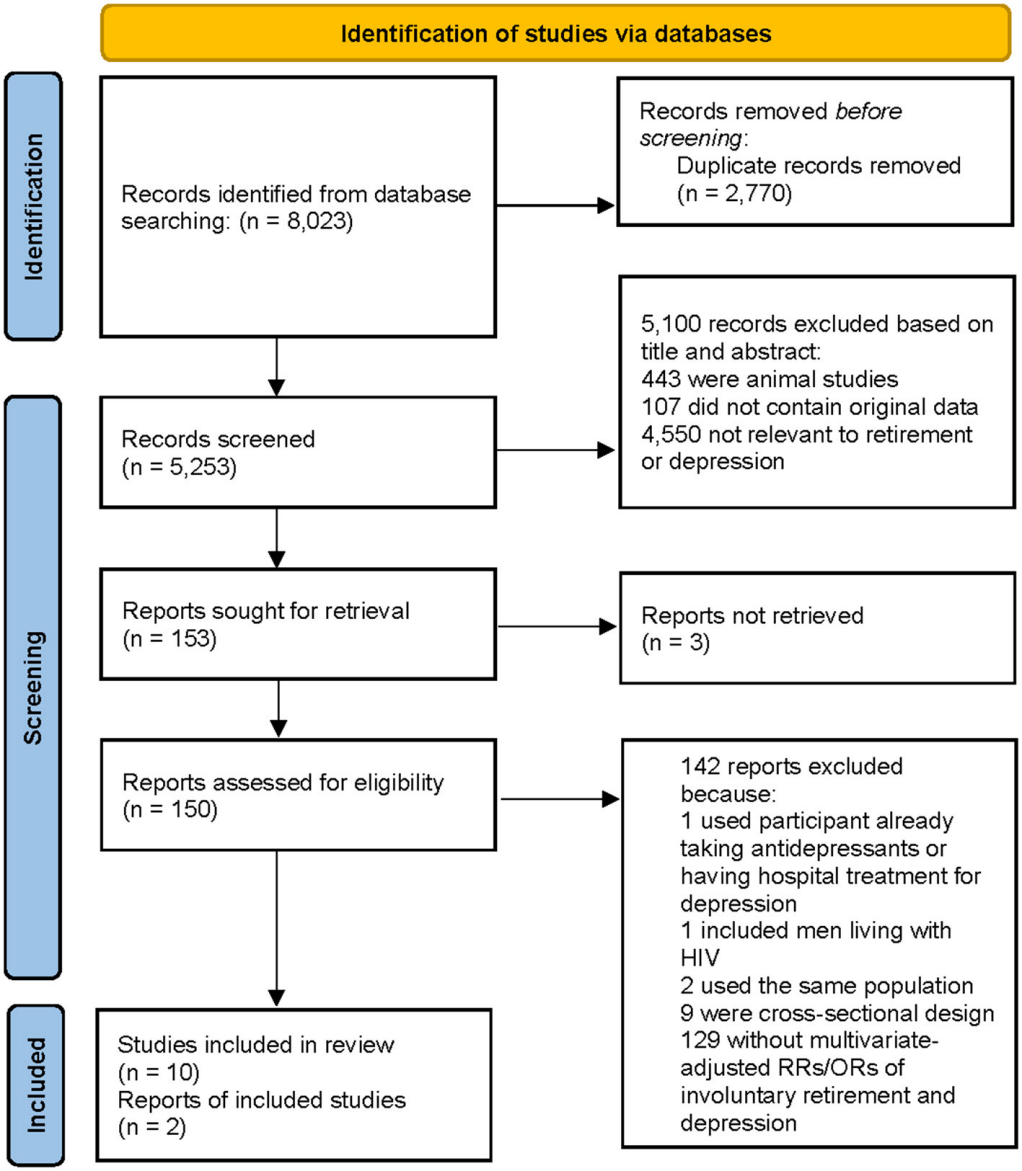
Flow of the literature.

We identified 8,023 articles by our literature search, of which 5,100 were excluded after review of titles or abstracts (Figure 1). Two additional articles were found in reference lists of retrieved studies. We reviewed 150 possibly relevant articles in full text. One article included participant already taking antidepressants or having hospital treatment for depression, one article included men living with HIV, two articles using the same population, nine cross-sectional designed articles, and 129 articles without multivariate-adjusted RRs/ORs concerning the relation between involuntary retirement and depression were excluded. Thus, 10 longitudinal studies from eight articles (5–12) were included in the analysis (Table 1).

**Table 1:** Characteristics of longitudinal studies on involuntary retirement and depression.

**Source, study and country (follow-up years)**	**Subjects, age and sex**	**Employment status measurement**	**Depression measurement**	**RR/OR (95%CI)**	**Adjustment for confounders**	**Study quality**
Karpansalo et al. (5); the Kuopio ischaemic heart disease risk factor study (KIHD); Finland (16 years)	1,726; 51.8; M	The pension registers of the Social Insurance Institution	The HPL (Human Population Laboratory) depression score	1.43 (1.21–1.79)	Age, education, occupation, body mass index, alcohol consumption, smoking, maximal oxygen uptake, and chronic diseases at baseline	9
Harkonmäki et al. (6); the Health and Social Support Study; Finland (5 years)	8,817; 40–54; M/F	Questionnaire	The 21-item Beck Depression Inventory	4.23 (2.91–6.14)	Age, gender, low socioeconomic status, health-related risk behavior, depression and use of drugs for somatic diseases	9
Doshi et al. (7); the Health and Retirement Study; U.S. (10 years)	2,853; 53–58; M/F	The Health and Retirement Study survey	Eight items of the standard Center for Epidemiologic Studies Depression scale (CES-D)	Men 1.53 (1.15–2.04) Women 1.56 (1.20–2.03)	Age, race/ethnicity, marital status, education, medical conditions, activity of daily living limitations, instrumental activity of daily living limitations, housing value, non-housing value, weekly wage, health insurance benefits, social security eligibility, type of pension availability	9
Rice et al. (8); the English Longitudinal Study of Aging; U.K. (4 years)	1,693; ≥50; M/F	Questionnaire	Center for Epidemiological Studies Depression (CES-D) symptoms index	1.50 (1.06–2.15)	Age; gender; individual pension wealth; alcohol consumption; self-rated health and partner retirement	8
Park and Kang (9); the Korean Longitudinal Study of Aging (KLoSA); Korea (6 years)	6,706; ≥45; M/F	Questionnaire	The short-form (10-item) Center for Epidemiological Studies-Depression (CES-D10) scale	Male 1.31 (1.063–1.613) Female 1.584 (1.216–2.062)	Age, property, household income, perceived health status and medical disability	9
Abuladze et al. (10); the Survey of Health, Aging and Retirement in Europe (SHARE) 2011–2015; Estonia (4 years)	1,851; ≥53; M/F	Computer-assisted personal interviewing (CAPI)	The EURO-D scale	1.45 (0.95–2.21)	Age, gender, education, marital status, receiving assistance, employment status, income, activity limitations, smoking, alcohol use, physical activity, satisfaction with life, depressiveness, computer skills, activities	9
Abrams et al. (11); the Health and Retirement Study (1992–2016); U.S. (24 years)	10,421; 51–61; M/F	The Health and Retirement Study survey	Eight items of the standard Center for Epidemiologic Studies Depression scale (CES-D)	1.16 (1.01–1.33)	sociodemographic factors, economic factors, health at expectations, health declines between expectation and age 62, and marriage/partnership dissolution between expectations and age 62	9
Pan et al. (12); the China Health and Retirement Longitudinal Study (CHARLS) 2011–2015; China (4 years)	5,616; ≥45; M/F	Interview	The 10-item Center for Epidemiological Studies–Depression (CES-D10) scale	1.24 (0.97–1.58)	Year, age, gender, marital status, residency, household registration system status, geographical region, family size, education, socio-economic status quartile, and work type	8

*OR, odds ratio; RR, relative risk; CI, confidence interval*.

All included studies had a longitudinal design. The duration of follow-up ranged from 4 to 24 years. Two studies (7, 9) included only women, three studies (5, 7, 9) included only men, and five studies (6, 8, 10–12) included men and women. With regard to the study region, four studies (5, 6, 8, 10) was conducted in Europe, three (7, 11) in America, and three (9, 12) in Asia. One study (5) measured employment status by record linkage, and other studies (6–12) used questionnaires. Seven studies (7–9, 11, 12) used Center for Epidemiologic Studies Depression scale to measure depressive symptoms, and the other three studies used EURO-D (10), Human Population Laboratory depression score (5), and Beck Depression Inventory (6), respectively. The major adjustment confounding factors included age, gender, education, race/ethnicity, and marital status. Quality assessment showed that the Newcastle-Ottawa score of each study was not <8, indicating that the methodological quality was generally good (Table S2).

### Involuntary Retirement and Depression

**Figure 2:**
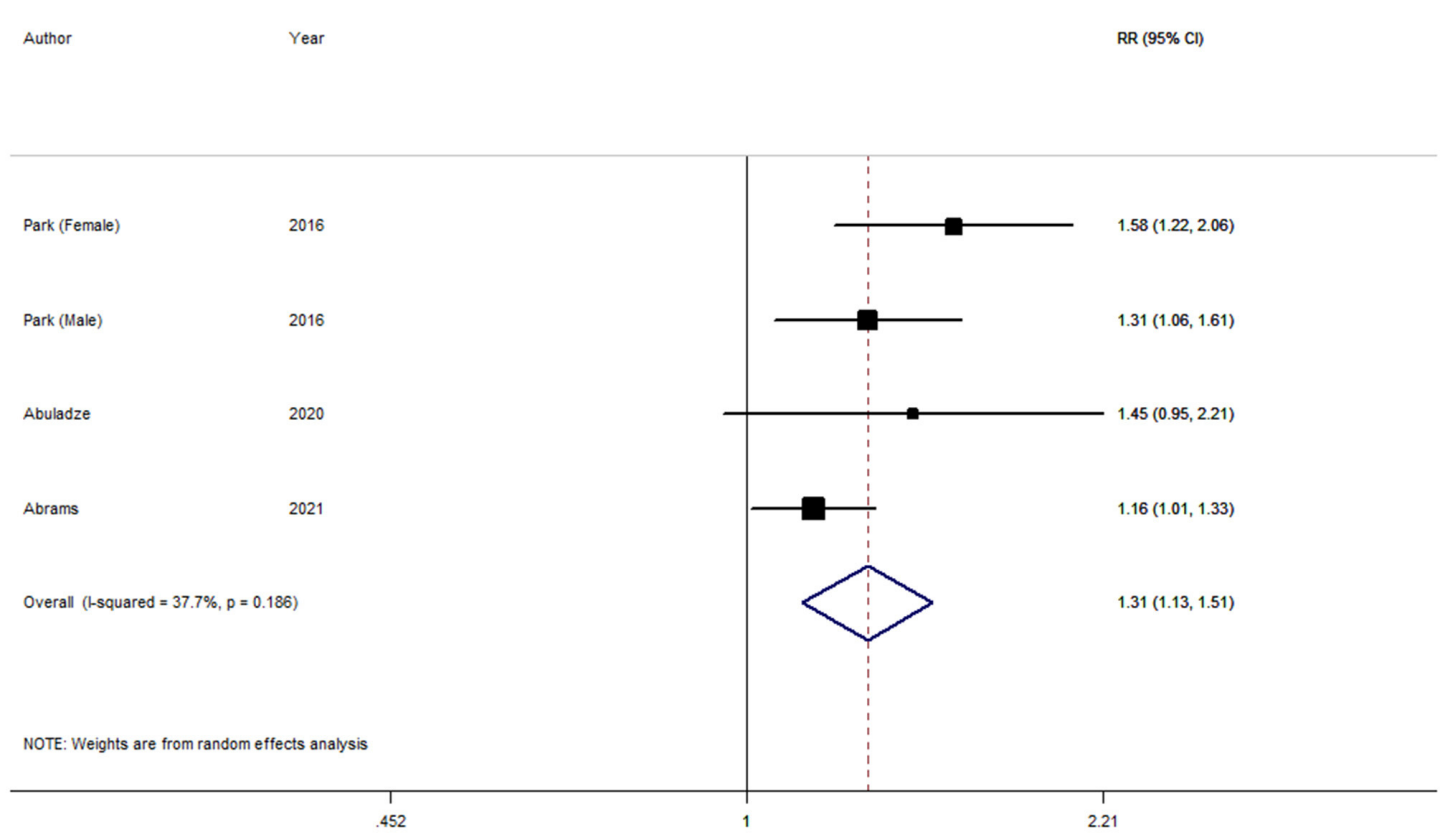
Forest plot for the pooled relative risk of depression.

Four longitudinal studies (9–11) involving 14,604 participants were included in the involuntary retirement and depression meta-analysis. Three studies (9, 11) showed a significant association between involuntary retirement and depression; while the other one study (10) indicated no relation between them. The pooled RR of overall data was 1.31 (95% CI, 1.13–1.51; *P* = 0) for the unexpectedly retired vs. employed, with low heterogeneity (*I*^2^ = 37.7 %, P_heterogeneity_ = 0.186) (Figure 2).

### Depression and Involuntary Retirement

**Figure 3:**
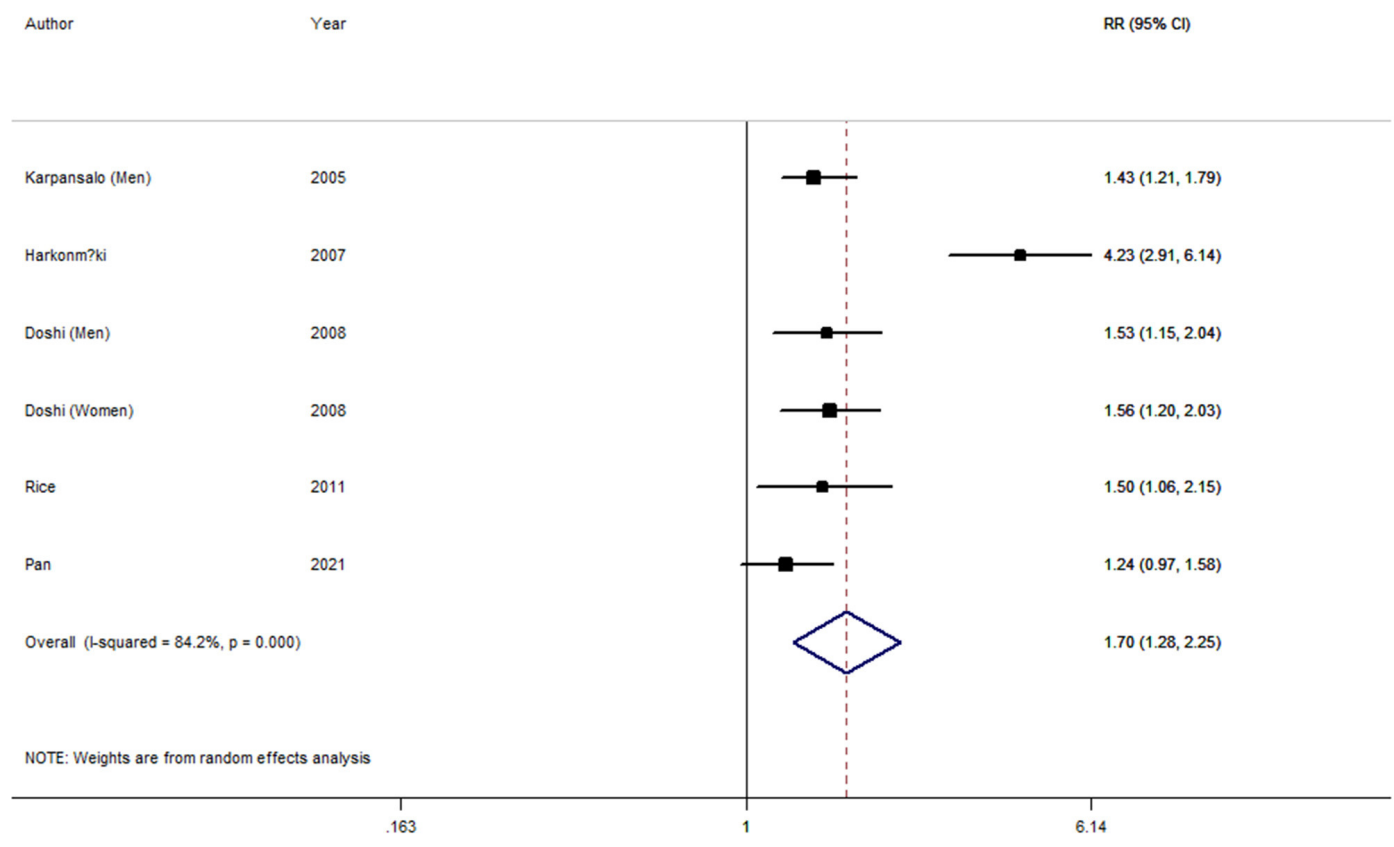
Forest plot for the pooled relative risk of involuntary retirement.

Six longitudinal studies (5–8, 12) involving 26,822 participants were included in the depression and involuntary retirement meta-analysis. Five studies (5–8) showed a significant association between depression and involuntary retirement; while the other one study (12) indicated no relation between them. The overall result indicated that depression was significantly associated with involuntary retirement (RR, 1.70; 95% CI, 1.28–2.25; *I*^2^ = 84.2%, *P*_heterogeneity_ = 0) (Figure 3).

### Meta-Regression and Subgroup Analyses

Low heterogeneity (*I*^2^ = 37.7 %, *P*_heterogeneity_ = 0.186) among all included studies was demonstrated for involuntary retirement and depression. *P*-values of univariate meta-regression analysis with the covariates of study region, number of participants and follow-up years were 0.212, 0.190, and 0.199, respectively.

As seen in Figure 3, high heterogeneity (*I*^2^ = 84.2%, *P*_heterogeneity_ = 0) was found for depression and involuntary retirement. *P*-values of univariate meta-regression analysis with the covariates of study region, number of participants and follow-up years were 0.949, 0.108, and 0.629, respectively.

Tables 2, 3 showed the results from subgroup analyses. The associations between involuntary retirement and depression did not differ substantially by study location and number of participants. For example, the pooled RRs for depression were consistent for studies conducted in Asia (RR, 1.42; 95% CI, 1.18–1.70). When we stratified studies by different number of participants, the pooled RRs of involuntary retirement were 1.45 (95% CI 1.22–1.72) for studies with subjects <4,000. For studies using Center for Epidemiologic Studies Depression scale to measure depressive symptoms, the pooled RRs were 1.30 (95% CI 1.10–1.54) for depression and 1.43 (95% CI 1.25–1.65) for involuntary retirement, respectively.

**Table 2:** Subgroup analyses of involuntary retirement and depression.

**Subgroup**	**No. of studies**	**RR (95%CI)**	**Heterogeneity**	
			***I*^2^ (%)**	***P*-value**
Location				
Asia	2	1.42 (1.18–1.70)	18.3	0.269
Number of participants				
<4,000	3	1.41 (1.21–1.65)	0	0.538
Depression measurement				
CES-D	3	1.30 (1.10–1.54)	54.6	0.012

*CES-D, Center for epidemiologic studies depression scale*.

**Table 3:** Subgroup analyses of depression and involuntary retirement.

**Subgroup**	**No. of studies**	**RR (95%CI)**	**Heterogeneity**	
			***I*^2^ (%)**	***P*-value**
Location				
Europe	3	2.06 (1.19–3.88)	92.4	0
America	2	1.55 (1.27–1.88)	0	0.92
Number of participants				
≥4,000	4	1.85 (1.18–2.91)	90.0	0
<4,000	2	1.45 (1.22–1.72)	0	0.817
Depression measurement				
CES-D	4	1.43 (1.25–1.65)	0	0.569

*CES-D, Center for epidemiologic studies depression scale*.

### Sensitivity Analysis

One study (6) was found to be the key contributor to this high between-study heterogeneity for depression and early retirement by the leave-one-out sensitivity analysis. After further excluding this study, no heterogeneity (*I*^2^ = 0, *P*_heterogeneity_ = 0.732) was found, and the pooled RR was 1.43 (95% CI 1.28–1.60).

### Publication Bias

**Figure 4:**
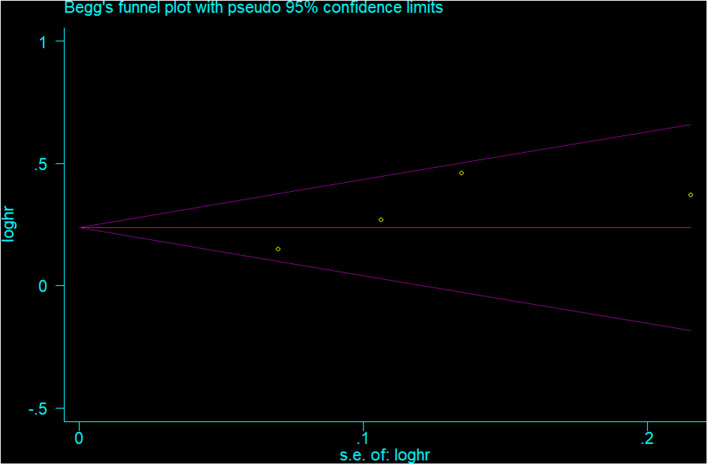
Funnel plot for the publication bias of depression analysis.

**Figure 5:**
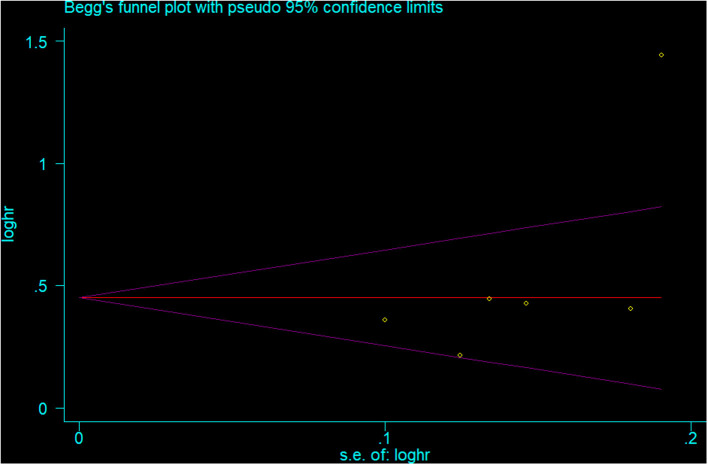
Funnel plot for the publication bias of involuntary retirement analysis.

The visual inspection of funnel plots (Figure 4 for depression and Figure 5 for involuntary retirement), Begg's rank correlation test (*p* = 0.308 for depression and *p* = 0.260 for involuntary retirement), and Egger's linear regression test (*p* = 0.180 for depression and *p* = 0.167 for involuntary retirement) showed no evidence of publication bias for the analysis between involuntary retirement and depression.

## Discussion

This study provides for the first time a meta-analysis of longitudinal-only studies examining the bidirectional association between involuntary retirement and depression. The meta-analysis of longitudinal studies including 14,604 participants for involuntary retirement and depression and 26,822 participants for depression and involuntary retirement identified that involuntary retirement was significantly associated with increased risk of depression and depression was an independent predictor of involuntary retirement in adults.

Recently, several studies researched the relation of retirement and depression (depressive symptoms scores). Findings from the present study were in agreement with a meta-analysis that used the standardized mean difference (d) as a measure of effect size and found involuntary retirement was associated with more depressive symptoms (*d* = 0.180, 95% CI 0.061–0.299) (18). Other reviews also found that people from developed Asian countries and lower socio-economic groups experienced a decline in mental health after retirement (19, 20).

The mechanisms underlying the association between involuntary retirement and depression are still not fully understood. One underlying explanation for our findings is that involuntary retirement may lead to changes in life patterns and social support, which has been important predictor of depression (21, 22). Otherwise, depression can also lead to involuntary retirement by affecting physical and social abilities (23, 24). Hence, retirees (depressed people) with disordered social intercourse and changed life routine are more likely to be depressed (retired). Further studies in understanding the underlying biological mechanisms linking involuntary retirement and depression are warranted.

Between-study heterogeneity occurs frequently in meta-analysis (13). In this study, low heterogeneity (*P*_heterogeneity_ = 0.186) was found in depression analysis, whereas high heterogeneity (*P*_heterogeneity_ = 0) was found in involuntary retirement analysis. Thus, we used meta-regression and the leave-one-out sensitivity analysis that aimed to explore the potentially important causes of the between-study heterogeneity. Univariate meta-regression showed that no abovementioned covariate was found to influence between-study heterogeneity. In our subgroup analyses by study region and number of participants, the between-study heterogeneity was decreased and the associations did not substantially change. One study (6) was found to be the key contributor to the high between-study heterogeneity of involuntary retirement analysis by the leave-one-out sensitivity analysis. When we excluded this study, there was no heterogeneity (*I*^2^ = 0%). After reducing the between-study heterogeneity, the results were found to be consistent with the one based on all studies, indicating that our results were stable and reliable.

A major strength of this study was the large number of participants included from longitudinal studies, allowing a much greater possibility of reasonable conclusions and investigating a potential causal relationship between involuntary retirement and depression. Second, all included studies had adjusted for potential confounders, increasing the credibility of the results. Third, the relationship between involuntary retirement and depression risk did not substantially change in sensitivity and subgroup analyses. Fourth, we found little evidence of publication bias in this meta-analysis, which indicated that our results were not affected by small-study effects.

Our results must be interpreted in light of the following limitations. First, although one study (10) considered satisfaction with life, and two studies (9) adjusted for disability, other psychological (personality traits and adaptability) and social (social networks and social activities) factors were not included, which may play a significant role in the presence or absence of depression in retirement age. Second, the limited information provided in the included studies precluded the possibility of subgroup analyses by different sex and income levels. Third, as only one study (5) used record linkage measures for employment status, the estimation of the true association between involuntary retirement and depression could be influenced by misclassification. Fourth, depression is considered a chronic, recurrent, remitting and continuous phenomenon. For included studies, the duration of follow-up had a wide range of 4–24 years; hence, the presence of previous depressive episodes could have an effect on the outcomes.

In summary, results from this meta-analysis indicate that there may be mutual causal relationship between involuntary retirement and depression in adults. More large studies with different gender and income levels are needed to identify the relation of involuntary retirement and depression in different groups of people and investigate the underlying biological mechanisms.

## Data Availability Statement

The original contributions presented in the study are included in the article/Supplementary Material, further inquiries can be directed to the corresponding author/s.

## Author Contributions

LZ and HZ conceived the study, participated in its design, and coordination. LZ and JW carried out the literature searching, data extraction, and quality assessment. YL and HZ were involved in the interpretation of the data, drafting the manuscript, and revising it critically for important intellectual content. All authors contributed to the article and approved the submitted version.

## Conflict of Interest

The authors declare that the research was conducted in the absence of any commercial or financial relationships that could be construed as a potential conflict of interest.

## Publisher's Note

All claims expressed in this article are solely those of the authors and do not necessarily represent those of their affiliated organizations, or those of the publisher, the editors and the reviewers. Any product that may be evaluated in this article, or claim that may be made by its manufacturer, is not guaranteed or endorsed by the publisher.
