# Supplementary material for: Involuntary Retirement and Depression Among Adults: A Systematic Review and Meta-Analysis of Longitudinal Studies
Source: Front Psychiatry. 2022 Feb 4;13:747334. doi: 10.3389/fpsyt.2022.747334 (PMC8854640; doi:10.3389/fpsyt.2022.747334)
Supplement: Supplementary file 1 [file Table_1.DOCX]

Supplementary table 1

| Databases | Search Details | Results |
| --- | --- | --- |
| PubMed | ("retiral"[All Fields] OR "retirement"[MeSH Terms] OR "retirement"[All Fields] OR "retire"[All Fields] OR "retired"[All Fields] OR "retirements"[All Fields] OR "retiring"[All Fields] OR "retires"[All Fields]) AND ("depressed"[All Fields] OR "depression"[MeSH Terms] OR "depression"[All Fields] OR "depressions"[All Fields] OR "depression s"[All Fields] OR "depressive disorder"[MeSH Terms] OR ("depressive"[All Fields] AND "disorder"[All Fields]) OR "depressive disorder"[All Fields] OR "depressivity"[All Fields] OR "depressive"[All Fields] OR "depressively"[All Fields] OR "depressiveness"[All Fields] OR "depressives"[All Fields]) AND ("english"[Language] OR "chinese"[Language]) | 2228 |
| Web of Science | ((ALL=(retirement)) AND ALL=(depression)) AND ((LA=(English)) OR LA=(Chinese)) | 1867 |
| Embase | (retirement and depression).mp. [mp=title, abstract, heading word, drug trade name, original title, device manufacturer, drug manufacturer, device trade name, keyword heading word, floating subheading word, candidate term word] | 2344 |
| ScienceDirect | Title, abstract, keywords: retirement AND depression | 324 |
| Wanfang | THEME= retirement AND THEME= depression | 1007 |
| VIP | U= 'retirement' AND U= 'depression' | 253 |
