# Supplementary material for: Involuntary Retirement and Depression Among Adults: A Systematic Review and Meta-Analysis of Longitudinal Studies
Source: Front Psychiatry. 2022 Feb 4;13:747334. doi: 10.3389/fpsyt.2022.747334 (PMC8854640; doi:10.3389/fpsyt.2022.747334)
Supplement: Supplementary file 2 [file Table_2.DOC]

Supplementary table 2 Quality assessment of longitudinal studies included in the meta-analysis^1^

| First author, year | Representativeness of the exposed cohort | Selection of the unexposed cohort | Ascertainment of exposure | Outcome of interest not present at start of study | Control for important factor or additional factor^2^ | Outcome assessment | Follow-up long enough for outcomes to occur^3^ | Adequacy of follow-up of cohorts^4^ |
| --- | --- | --- | --- | --- | --- | --- | --- | --- |
| Karpansalo et al. (2005) | * | * | * | * | ** | * | * | * |
| Harkonmäki et al. (2007) | * | * | * | * | ** | * | * | * |
| Doshi et al. (2008) | * | * | * | * | ** | * | * | * |
| Rice et al. (2011) | * | * | * |  | ** | * | * | * |
| Park et al. (2016) | * | * | * | * | ** | * | * | * |
| Abuladze et al. (2020) | * | * | * | * | ** | * | * | * |
| Abrams et al. (2021) | * | * | * | * | ** | * | * | * |
| Pan et al. (2021) | * | * | * |  | ** | * | * | * |

^1^ A study could be awarded a maximum of one star for each item except for the item Control for important factor or additional factor.

^2^ A maximum of 2 stars could be awarded for this item. Studies that controlled for age or gender received one star, whereas studies that controlled for other important confounders such as chronic health conditions received an additional star.

^3^ A cohort study with a follow-up time >3 years was assigned one star.

^4^ A cohort study with a follow-up rate >70% was assigned one star.
